# Supplementary material for: TbpB-based oral mucosal vaccine provides heterologous protection against Glässer’s disease caused by different serovars of Spanish field isolates of Glaesserella parasuis
Source: Porcine Health Manag. 2024 Nov 28;10:57. doi: 10.1186/s40813-024-00404-7 (PMC11606180; doi:10.1186/s40813-024-00404-7)
Supplement: Supplementary file 1 — Additional file 1: Tables S1–S3. Scores used for the evaluation of clinical signs, inflammatory and vascular macroscopic lesions, and histopathological lesions. File format: .docx. [file 40813_2024_404_MOESM1_ESM.docx]

**Table S1.** Detailed explanation of the clinical scores used for the daily monitoring of pigs during challenged with *Glaesserella parasuis*.

| **Clinical Sign** | **Score** | | | |
| --- | --- | --- | --- | --- |
|  | **0 = No clinical signs** | **1 = Mild clinical signs** | **2 = Moderate clinical signs** | **3 = Severe clinical signs** |
| **Apathy** | The pig exhibits normal activity, responds quickly to external stimuli (e.g., sounds or human presence), moves freely, and engages in typical behaviours such as feeding and social interaction. | Slight delay in responding to stimuli but still moves and feeds without significant issues. It may appear slightly less active than usual, though no overt signs of distress are evident. | The pig responds slowly to external stimuli, shows reduced interest in feeding or interacting with other pigs, and displays limited mobility.  Decrease in activity, with a tendency to lie down for extended periods. | Minimal response to external stimuli, with little or no movement and a marked reluctance to eat. The pig remains lying down for long periods, with very little interest in its surroundings. |
| **Lack of appetite** | The pig consumes food regularly with no signs of reduced interest. | Slight hesitation or delay before feeding, consuming less than usual. Most feed is eventually consumed. | Clear decrease in feed intake, consuming significantly less than normal.  It may approach the feed but only eat small amounts before losing interest. | No interest in feed, and does not consume any.  This complete lack of appetite is typically accompanied by other signs of illness or distress, such as weight loss or lethargy. |
| **Lameness** | The pig moves freely with no observable gait abnormalities. | Slight but noticeable gait irregularity. It may limp or favor one limb but is still weight-bearing on all legs.  Mobility is somewhat reduced, but the pig is still able to walk and stand with minor difficulties. | Favors one or more limbs and bears limited weight on the affected limb or limbs.  It moves with obvious discomfort, with more pronounced gait irregularity.  Mobility is markedly impaired, but the pig is still able to walk. | Struggles to bear the weight of the affected limb or limbs and may avoid using them altogether.  Movement is severely restricted. The pig may only walk short distances or may prefer to lie down most of the time. |
| **Incoordination** | The pig shows normal, smooth and coordinated movements. There are no signs of stumbling, unsteady gait or difficulty maintaining balance. | Slight instability or stumbling. Can still move effectively without falling. | Noticeable difficulties in coordinating movements, with frequent stumbling, swaying or difficulty maintaining a straight line when walking. The pig may have to adjust its posture to regain balance, but is still able to walk. | Unable to stand or walk due to severe incoordination. It may repeatedly collapse when attempting to move and cannot maintain balance or effectively coordinate its limbs. |
| **Dyspnea** | Normal, quiet, and regular breathing patterns. There are no signs of respiratory distress, such as increased respiratory rate or labored breathing. | Slight increase in respiratory rate or occasional signs of mild labored breathing. There may be a subtle increase in respiratory effort, but the pig remains relatively comfortable and shows no significant signs of distress. | Clear respiratory distress, such as a marked increase in respiratory rate, visible effort to breathe, occasional audible wheezing or coughing. The pig may appear uncomfortable and may show respiratory distress during physical activity. | Severe respiratory distress, characterized by very rapid and labored breathing with great effort, pronounced nasal flaring and possible cyanosis (bluish discoloration of the mucous membranes). The pig may be in a state of severe discomfort or panic, struggling to breathe and unable to perform normal activities. |
| **Joint inflammation** | The pig shows no visible swelling, redness or joint pain. Joint movement is normal, with no palpable warmth or tenderness in the joints. | Slight swelling or warmth in one or more joints, but the pig shows only minor discomfort.  Joint movement is largely unaffected, and a slight reluctance to move may be observed. | Clear swelling is observed in the affected joint or joints, often accompanied by slight redness and warmth. The pig shows moderate discomfort and reduced range of motion in the joint or joints, with visible signs of stiffness or lameness during movement. | The affected joint or joints show significant swelling, warmth and redness. The pig shows pronounced pain, reluctance to move, and marked reduction in joint function. There is marked stiffness or inability to fully bear weight on the affected limb or limbs. |

**Table S2.** Detailed explanation of the macroscopic inflammatory and vascular lesions scores used during the necropsy of pigs challenged with *Glaesserella parasuis*.

| **Lesion** | | **Score** | | | |
| --- | --- | --- | --- | --- | --- |
|  |  | **0 = No lesion** | **1 = Mild lesion** | **2 = Moderate lesion** | **3 = Severe lesion** |
| **Inflammatory** | **Arthritis** | No lesion. | Turbid fluid present in excess of normal joint fluid. | Small deposits/strands of fibrin with or without fluid. | Presence of abundant fibrin. |
|  | **Peritonitis, pericarditis and**  **pleuritis** | No lesion. | Turbid liquid in one or more body cavities. | Small fibrin deposits/threads with or without fluid in one or more body cavities. | Presence of abundant fibrin in one or more body cavities. |
| **Vascular** | **Congestion (kidneys, brain, liver and spleen)** | No lesion | Slight accumulation of blood is visible in the vessels of the affected organ.  The organ may appear slightly darker or more reddish than usual. | Clear evidence of distended blood vessels and noticeable darkening or reddening of the organ. | Marked distended blood vessels and significant darkening or purplish color of the affected organs |
|  | **Gallbladder edema** | No lesion | Slight accumulation of gelatinous fluid, which mildly separates the gallbladder wall from the hepatic parenchyma. | Moderate amount of gelatinous fluid causing noticeable separation of the gallbladder wall from the hepatic parenchyma. | Large amount of gelatinous fluid causing significant separation of the gallbladder wall from the hepatic parenchyma. |

**Table S3.** Detailed explanation of the histopathology scores used for the pigs challenged with *Glaesserella parasuis*.

| **Organ** | **Microscopic lesion** | | **Score** | | | | |
| --- | --- | --- | --- | --- | --- | --- | --- |
|  |  |  | **0 = No lesion** | **1 = Minor lesion** | **2 = Mild lesion** | **3 = Moderate lesion** | **4 = Severe lesion** |
| **Lungs** | **Interstitial pneumonia** | | No inflammatory lesion. | Low number of mononuclear  inflammatory  cells in  alveolar septa affecting only some lung lobules.  Very slight thickness of alveolar walls.  No narrowing of the alveolar spaces. | Evident presence of mononuclear  inflammatory  cells in  alveolar septa in some lung lobules.  Slight thickness of alveolar walls.  No narrowing of the alveolar spaces | Many mononuclear inflammatory cells in  alveolar septa in many lung lobules.  Evident  thickening of alveolar septa.  Evident narrowing of the alveolar spaces. | Large number of inflammatory cells in  alveolar septa in most lung lobules.  Marked  thickening of alveolar septa.  Significant narrowing of the alveolar spaces. |
|  | **Pleuritis** | | No inflammatory lesion. | Scarce (5-10) inflammatory cells. | Few (>10) to small groups of inflammatory cells. | Multifocal to diffuse evident inflammation. | Marked inflammation. |
|  | **Vascular disorders** | **Hemorrhages** | No presence of red blood cells outside the vessels. | Sporadic and focal presence of red blood cells outside the vessels. | Few and focal presence of red blood cells outside the vessels. | Diffuse and evident presence of red blood cells outside the vessels. | Diffuse and marked presence of red blood cells outside the vessels. |
|  |  | **Alveolar/Interstitial edema** | No presence of acidophilic material in interstitium and/or alveolar lumen | Sporadic and focal presence of acidophilic material in interstitium and/or alveolar lumen | Few and focal presence of acidophilic material in interstitium and/or alveolar lumen | Diffuse and evident presence of acidophilic material in interstitium and/or alveolar lumen | Diffuse and marked presence of acidophilic material in interstitium and/or alveolar lumen. |
| **Brain** | **Encephalitis**  **Meningitis**  **Meningoencephalitis**  **Ventriculitis** | | No inflammatory lesion. | Scarce (5-10) inflammatory cells. | Few (>10) to small groups of inflammatory cells. | Multifocal to diffuse evident inflammation. | Marked inflammation. |
| **Liver** | **Gallbladder edema** | | No presence of acidophilic material in gallbladder wall. | Sporadic and focal presence of acidophilic material in gallbladder wall. | Few and focal presence of acidophilic material in gallbladder wall. | Multifocal to diffuse presence of acidophilic material in gallbladder wall. | Diffuse and marked presence of acidophilic material in gallbladder wall. |
|  | **Liver congestion** | | No hepatic sinusoids affected. | Few hepatic sinusoids affected. | Some hepatic sinusoids affected. | Many hepatic sinusoids affected. | Most hepatic sinusoids affected. |
|  | **Hepatitis** | | No inflammatory lesion. | Scarce (5-10) inflammatory cells. | Few (>10) to small groups of inflammatory cells. | Multifocal to diffuse evident inflammation. | Marked inflammation. |
| **Spleen** | **Red pulp congestion** | | No splenic sinuses affected. | Few splenic sinuses affected. | Some splenic sinuses affected. | Many splenic sinuses affected. | Most splenic sinuses affected. |
